# (3-Methyl­benzo­nitrile-κ*N*)tetra­kis(μ-*N*-phenyl­acetamidato)-κ^4^ *N*:*O*;κ^4^ *O*:*N*-di­rhodium(II)(*Rh*—*Rh*)

**DOI:** 10.1107/S1600536813029838

**Published:** 2013-11-06

**Authors:** Cassandra T. Eagle, Nkongho Atem-Tambe, Kenneth K. Kpogo, Jennie Tan, Fredricka Quarshie

**Affiliations:** a Department of Chemistry, East Tennessee State University, PO Box 70695, Johnson City, TN 37614, USA

## Abstract

In the title compound, [Rh_2_(C_8_H_8_NO)_4_(C_8_H_7_N)], the four acetamidate ligands bridging the dirhodium core are arranged in a 2,2-*trans* manner. One Rh^II^ atom is five-coordinate, in a distorted pyramidal geometry, while the other is six-coord­in­ate, with a disorted octa­hedral geometry. For the six-coord­inate Rh^II^ atom, the axial nitrile ligand shows a non-linear Rh–nitrile coordination with an Rh—N—C bond angle of 166.4 (4)° and a nitrile N—C bond length of 1.138 (6) Å. Each unique Rh^II^ atom is coordinated by a *trans* pair of N atoms and a *trans* pair of O atoms from the four acetamide ligands. The N_eq_—Rh—Rh—O_eq_ torsion angles on the acetamide bridge varies between 12.55 (11) and 14.04 (8)°. In the crystal, the 3-methyl­benzo­nitrile ring shows a π–π inter­action with an inversion-related equivalent [inter­planar spacing = 3.360 (6) Å]. A phenyl ring on one of the acetamide ligands also has a face-to-face π–π inter­action with an inversion-related equivalent [inter­planar spacing = 3.416 (5) Å].

## Related literature

For the synthesis and structures of three related compounds, see Eagle *et al.* (2000 ▶, 2012 ▶, 2013 ▶).
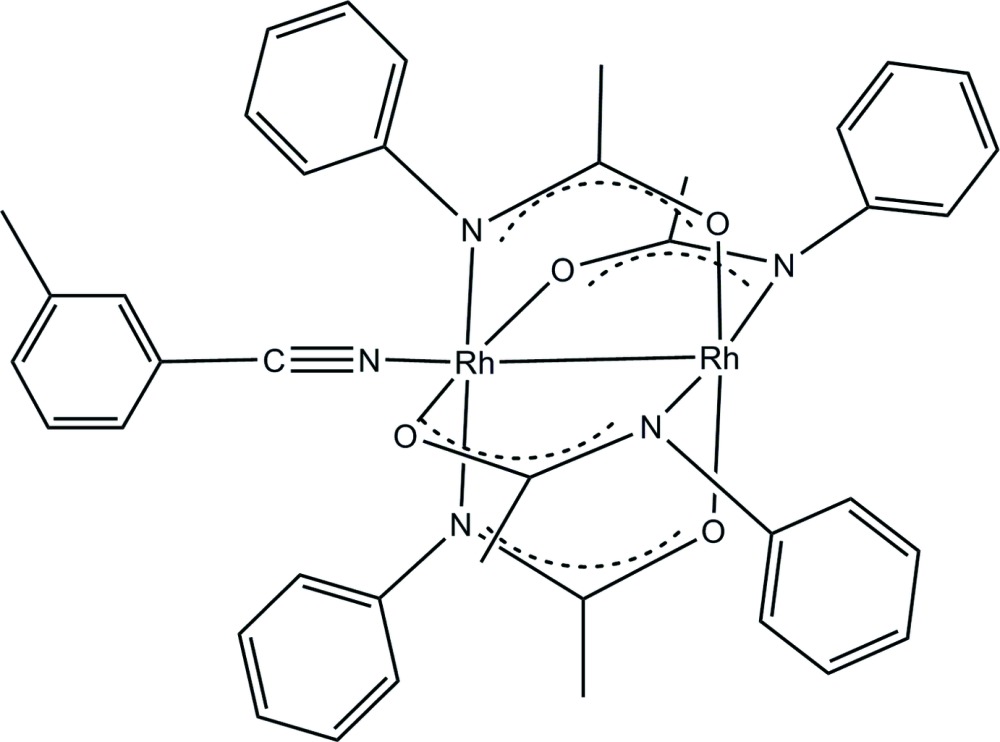


## Experimental

### 

#### Crystal data

[Rh_2_(C_8_H_8_NO)_4_(C_8_H_7_N)]
*M* *_r_* = 859.58
Triclinic, 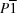

*a* = 11.7109 (13) Å
*b* = 13.0181 (14) Å
*c* = 13.3980 (14) Å
α = 72.337 (5)°
β = 66.780 (5)°
γ = 82.742 (6)°
*V* = 1788.6 (3) Å^3^
*Z* = 2
Mo *K*α radiation
μ = 0.97 mm^−1^
*T* = 298 K
0.16 × 0.08 × 0.07 mm

#### Data collection

Rigaku XtaLAB mini diffractometer
Absorption correction: multi-scan (*REQAB*; Rigaku, 1998 ▶) *T*_min_ = 0.774, *T*_max_ = 0.934
18460 measured reflections
8156 independent reflections
5635 reflections with *I* > 2σ(*I*)
*R*_int_ = 0.065

#### Refinement

*R*[*F* ^2^ > 2σ(*F* ^2^)] = 0.049
*wR*(*F* ^2^) = 0.102
*S* = 1.04
8156 reflections
465 parameters
H-atom parameters constrained
Δρ_max_ = 0.73 e Å^−3^
Δρ_min_ = −0.86 e Å^−3^

### 

Data collection: *CrystalClear-SM Auto* (Rigaku, 2011 ▶); cell refinement: *CrystalClear-SM Auto*; data reduction: *CrystalClear-SM Auto*; program(s) used to solve structure: *SHELXS97* (Sheldrick, 2008 ▶); program(s) used to refine structure: *SHELXL97* (Sheldrick, 2008 ▶) and *SHELXL2013* (Sheldrick, 2008 ▶); molecular graphics: *CrystalStructure* (Rigaku, 2010 ▶); software used to prepare material for publication: *CrystalStructure* and *Mercury* (Macrae *et al.*, 2008 ▶).

## Supplementary Material

Crystal structure: contains datablock(s) I, New_Global_Publ_Block. DOI: 10.1107/S1600536813029838/pk2497sup1.cif

Structure factors: contains datablock(s) I. DOI: 10.1107/S1600536813029838/pk2497Isup2.hkl

Additional supplementary materials:  crystallographic information; 3D view; checkCIF report

## Figures and Tables

**Fig. 1.**
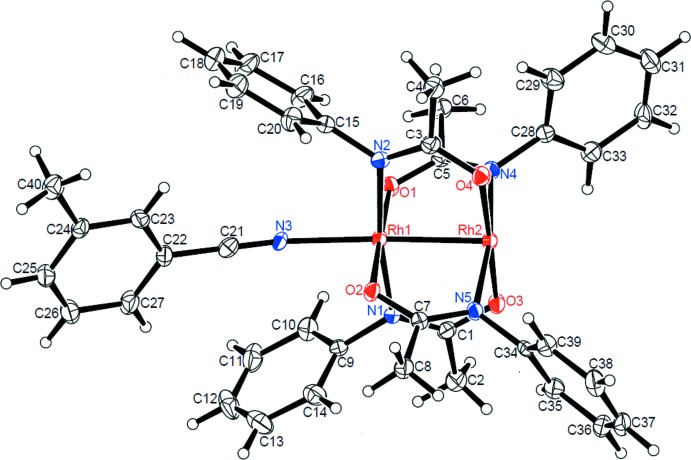
An ellipsoid plot of title compound showing 30% probability ellipsoids. Hydrogen atoms are drawn as small spheres.

**Fig. 2.**
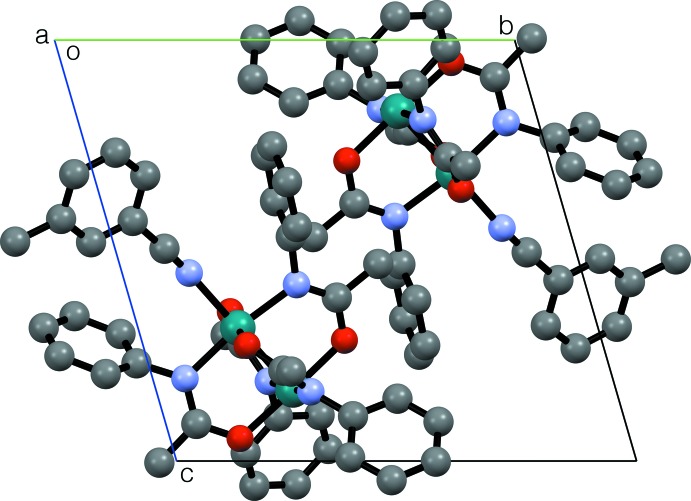
Packing diagram of title compound viewed down the a axis.

## supplementary crystallographic information

### 1. Comment

Previous papers report the structures of the related complexes 2,2-*trans*-[Rh_2_(N(C_6_H_5_)COCH_3_)_4_]·2NCC_6_H_5_ (2) (Eagle *et al.*, 2000), 2,2-*trans*-[Rh_2_(N(C_9_H_11_)COCH_3_]_4_·2NCC_6_H_5_ (3) (Eagle *et al.*, 2012) and 2,2-*cis*-[Rh_2_(N(C_6_H_5_)COCH_3_)_4_]·2NCC_6_H_5_ (4) (Eagle *et al.*, 2013). The numbering scheme of the title compound is adapted from that of compound 2.

The axial rhodium-nitrogen-carbon bond angle for the title compound, 1, of 166.4 (4)° (see Fig.1) is distinctly non-linear, and contrasts with those found in compound 2 [178.5 (5)° and 169.3 (5)°], compound 3 (180°; imposed by space group symmetry) and compound 4 [167.14 (15)°]. The axial carbon—nitrogen bond length in 1 is 1.138 (6) Å which is comparable to corresponding distances found in 2 [1.135 (8) Å and 1.145 (8) Å] as well as 4 [1.135 (3) Å] and slightly longer than 3 [1.106 (6) Å]. Compound 1 has torsion angles around each acetamide bridge varying between 12.55 (11)° and 14.04 (8)°. These can be compared to the range of 9.03° and 11.89° in 2, 1.12 (9)° in 3 and the range of 1.62 (4)° and 1.78 (4)° in 4. A packing diagram of the structure is shown in Fig. 2.

The infrared absorption spectrum of compound 1 showed a band at 2310.72 cm^-1^ attributable to the carbon—nitrogen bond stretching mode. The corresponding band for uncomplexed 3-methyl benzonitrile appears at 2227.78 cm^-1^. This indicates that there is a shortening of the carbon—nitrogen bond and a stronger σ-interaction to the rhodium metal compared to the π-back bonding which occurs upon complexation with *trans-*tetrakis(µ-*N*-phenylacetamidato)-κ^4^*N*:*O*;κ^4^*O*:*N*-dirhodium(II).

### 2. Experimental

Approximately 20 mg of the *trans-*tetrakis(µ-*N*-phenylacetamidato)-κ^4^*N*:*O*;κ^4^*O*:*N*-dirhodium(II) was dissolved in 5 ml dichloromethane. 6.5 µ*L* of neat 3-methyl benzonitrile was then added to this solution, *via* a gas tight syringe, turning the solution from a forest green color to dark blue. Crystals grew over a two week period *via* vapor diffusion. From the structure determination, compound 1 is an adduct of *trans-*tetrakis(µ-*N*-phenylacetamidato)-κ^4^*N*:*O*;κ^4^*O*:*N*-dirhodium(II) with 3-methyl benzonitrile in one axial site.

### 3. Refinement

H-atoms were included in calculated positions with C—H = 0.93 - 0.96 Å and included as riding contributions with isotropic displacement parameters 1.2 - 1.5 times those of the attached atom.

### Crystal data

**Table:**

[Rh_2_(C_8_H_8_NO)_4_(C_8_H_7_N)]	*Z* = 2
*M**_r_* = 859.58	*F*(000) = 872
Triclinic, *P*1	*D*_x_ = 1.596 Mg m^−^^3^
*a* = 11.7109 (13) Å	Mo *K*α radiation, λ = 0.71075 Å
*b* = 13.0181 (14) Å	Cell parameters from 14162 reflections
*c* = 13.3980 (14) Å	θ = 3.0–27.6°
α = 72.337 (5)°	µ = 0.97 mm^−^^1^
β = 66.780 (5)°	*T* = 298 K
γ = 82.742 (6)°	Block, blue
*V* = 1788.6 (3) Å^3^	0.16 × 0.08 × 0.07 mm

### Data collection

**Table:**

Rigaku XtaLAB mini diffractometer	8156 independent reflections
Radiation source: fine-focus sealed X-ray tube	5635 reflections with *I* > 2σ(*I*)
Graphite Monochromator monochromator	*R*_int_ = 0.065
Detector resolution: 6.827 pixels mm^-1^	θ_max_ = 27.5°, θ_min_ = 3.0°
ω scans	*h* = −15→15
Absorption correction: multi-scan (*REQAB*; Rigaku, 1998)	*k* = −16→16
*T*_min_ = 0.774, *T*_max_ = 0.934	*l* = −17→17
18460 measured reflections	

### Refinement

**Table:**

Refinement on *F*^2^	0 constraints
Least-squares matrix: full	Primary atom site location: structure-invariant direct methods
*R*[*F*^2^ > 2σ(*F*^2^)] = 0.049	Hydrogen site location: inferred from neighbouring sites
*wR*(*F*^2^) = 0.102	H-atom parameters constrained
*S* = 1.04	*w* = 1/[σ^2^(*F*_o_^2^) + (0.0345*P*)^2^ + 1.6123*P*] where *P* = (*F*_o_^2^ + 2*F*_c_^2^)/3
8156 reflections	(Δ/σ)_max_ = 0.022
465 parameters	Δρ_max_ = 0.73 e Å^−^^3^
0 restraints	Δρ_min_ = −0.86 e Å^−^^3^

### Special details

**Table:**

Geometry. The 3-methyl benzonitrile rings (composed of carbon atoms C22–27) are π- stacked with inversion related symmetry equivalents (symmetry code: 2 - *x*, 2 - *y*, 1 - *z*). The interplanar spacing is 3.360 (6) Å. One of the phenyl rings on the acetamide ligand (composed of carbon atoms C34—C39) has face to face π-π interaction with its symmetry equivalent (symmetry code:2 - *x*, 1 - *y*, -*z*). The interplanar spacing is 3.416 (5) Å.All e.s.d.'s (except the e.s.d. in the dihedral angle between two l.s. planes) are estimated using the full covariance matrix. The cell e.s.d.'s are taken into account individually in the estimation of e.s.d.'s in distances, angles and torsion angles; correlations between e.s.d.'s in cell parameters are only used when they are defined by crystal symmetry. An approximate (isotropic) treatment of cell e.s.d.'s is used for estimating e.s.d.'s involving l.s. planes.
Refinement. Refinement was performed using all reflections. The weighted *R*-factor (*wR*) and goodness of fit (*S*) are based on *F*^2^. *R*-factor (gt) are based on *F*. The threshold expression of *F*^2^ > 2.0 σ(*F*^2^) is used only for calculating *R*-factor (gt).

### Fractional atomic coordinates and isotropic or equivalent isotropic displacement parameters (Å^2^)

**Table:**

	*x*	*y*	*z*	*U*_iso_*/*U*_eq_	
Rh1	0.75889 (3)	0.78294 (3)	0.31656 (3)	0.02306 (10)	
Rh2	0.78739 (3)	0.70205 (3)	0.16908 (3)	0.02409 (10)	
O1	0.5727 (3)	0.7891 (2)	0.3547 (3)	0.0289 (7)	
O2	0.9463 (3)	0.7741 (2)	0.2746 (3)	0.0278 (7)	
O3	0.7241 (3)	0.5591 (2)	0.2870 (3)	0.0299 (7)	
O4	0.8450 (3)	0.8478 (2)	0.0536 (2)	0.0296 (7)	
N1	0.7374 (3)	0.6280 (3)	0.4193 (3)	0.0248 (8)	
N2	0.7779 (3)	0.9298 (3)	0.1961 (3)	0.0281 (8)	
N3	0.7472 (3)	0.8541 (3)	0.4466 (3)	0.0269 (8)	
N4	0.6066 (3)	0.7502 (3)	0.1894 (3)	0.0262 (8)	
N5	0.9663 (3)	0.6645 (3)	0.1648 (3)	0.0264 (8)	
C1	0.7162 (4)	0.5487 (4)	0.3882 (4)	0.0300 (11)	
C2	0.6808 (5)	0.4375 (4)	0.4681 (4)	0.0438 (13)	
H2A	0.7346	0.3850	0.4345	0.053*	
H2B	0.5963	0.4234	0.4829	0.053*	
H2C	0.6889	0.4334	0.5378	0.053*	
C3	0.8188 (4)	0.9336 (4)	0.0883 (4)	0.0284 (10)	
C4	0.8350 (5)	1.0383 (4)	−0.0037 (4)	0.0369 (12)	
H4A	0.8235	1.0262	−0.0664	0.044*	
H4B	0.9171	1.0653	−0.0284	0.044*	
H4C	0.7748	1.0900	0.0250	0.044*	
C5	0.5350 (4)	0.7823 (4)	0.2789 (4)	0.0281 (10)	
C6	0.4014 (4)	0.8139 (4)	0.2994 (4)	0.0358 (12)	
H6A	0.3971	0.8868	0.2555	0.043*	
H6B	0.3569	0.8085	0.3782	0.043*	
H6C	0.3650	0.7665	0.2776	0.043*	
C7	1.0111 (4)	0.7110 (4)	0.2149 (4)	0.0280 (10)	
C8	1.1421 (4)	0.6945 (4)	0.2126 (4)	0.0351 (11)	
H8A	1.1402	0.6810	0.2878	0.042*	
H8B	1.1900	0.7580	0.1645	0.042*	
H8C	1.1795	0.6339	0.1841	0.042*	
C9	0.7291 (4)	0.6104 (3)	0.5329 (4)	0.0281 (10)	
C10	0.6180 (5)	0.6224 (4)	0.6171 (4)	0.0441 (13)	
H10	0.5458	0.6365	0.6018	0.053*	
C11	0.6128 (6)	0.6134 (4)	0.7251 (5)	0.0527 (15)	
H11	0.5371	0.6204	0.7821	0.063*	
C12	0.7197 (7)	0.5942 (5)	0.7474 (5)	0.0609 (19)	
H12	0.7169	0.5881	0.8193	0.073*	
C13	0.8305 (7)	0.5842 (5)	0.6624 (5)	0.0583 (17)	
H13	0.9032	0.5727	0.6768	0.070*	
C14	0.8359 (5)	0.5908 (4)	0.5556 (5)	0.0414 (13)	
H14	0.9114	0.5820	0.4994	0.050*	
C15	0.7490 (4)	1.0255 (4)	0.2322 (4)	0.0305 (10)	
C16	0.6271 (5)	1.0417 (4)	0.3019 (4)	0.0395 (12)	
H16	0.5658	0.9927	0.3200	0.047*	
C17	0.5971 (5)	1.1305 (4)	0.3442 (5)	0.0503 (15)	
H17	0.5160	1.1403	0.3917	0.060*	
C18	0.6871 (6)	1.2047 (4)	0.3164 (5)	0.0501 (15)	
H18	0.6666	1.2651	0.3435	0.060*	
C19	0.8053 (6)	1.1884 (4)	0.2493 (5)	0.0493 (14)	
H19	0.8658	1.2382	0.2311	0.059*	
C20	0.8390 (5)	1.0991 (4)	0.2067 (4)	0.0398 (12)	
H20	0.9212	1.0890	0.1617	0.048*	
C21	0.7660 (4)	0.8925 (4)	0.5043 (4)	0.0326 (11)	
C22	0.8002 (4)	0.9440 (4)	0.5702 (4)	0.0311 (11)	
C23	0.8453 (4)	1.0479 (4)	0.5197 (4)	0.0327 (11)	
H23	0.8460	1.0844	0.4480	0.039*	
C24	0.8895 (4)	1.0983 (4)	0.5750 (4)	0.0307 (11)	
C25	0.8888 (5)	1.0417 (4)	0.6811 (4)	0.0390 (12)	
H25	0.9207	1.0734	0.7182	0.047*	
C26	0.8411 (5)	0.9385 (4)	0.7323 (4)	0.0431 (13)	
H26	0.8390	0.9025	0.8045	0.052*	
C27	0.7965 (5)	0.8882 (4)	0.6777 (4)	0.0412 (13)	
H27	0.7649	0.8187	0.7122	0.049*	
C28	0.5586 (4)	0.7518 (4)	0.1057 (4)	0.0281 (10)	
C29	0.5650 (5)	0.8446 (4)	0.0197 (5)	0.0427 (13)	
H29	0.6023	0.9058	0.0147	0.051*	
C30	0.5159 (5)	0.8464 (5)	−0.0588 (5)	0.0469 (14)	
H30	0.5182	0.9099	−0.1151	0.056*	
C31	0.4634 (5)	0.7554 (5)	−0.0555 (5)	0.0484 (15)	
H31	0.4311	0.7571	−0.1092	0.058*	
C32	0.4600 (5)	0.6627 (5)	0.0287 (5)	0.0420 (13)	
H32	0.4254	0.6008	0.0318	0.050*	
C33	0.5076 (4)	0.6602 (4)	0.1093 (4)	0.0359 (11)	
H33	0.5051	0.5968	0.1657	0.043*	
C34	1.0412 (4)	0.5882 (4)	0.1066 (4)	0.0252 (10)	
C35	1.0073 (4)	0.4806 (4)	0.1482 (4)	0.0345 (11)	
H35	0.9357	0.4582	0.2121	0.041*	
C36	1.0814 (5)	0.4059 (4)	0.0934 (4)	0.0404 (13)	
H36	1.0590	0.3337	0.1221	0.048*	
C37	1.1860 (4)	0.4368 (5)	−0.0012 (4)	0.0398 (13)	
H37	1.2356	0.3860	−0.0357	0.048*	
C38	1.2171 (5)	0.5446 (5)	−0.0452 (5)	0.0454 (14)	
H38	1.2864	0.5668	−0.1113	0.054*	
C39	1.1457 (4)	0.6205 (4)	0.0088 (4)	0.0356 (11)	
H39	1.1680	0.6928	−0.0209	0.043*	
C40	0.9357 (5)	1.2124 (4)	0.5211 (5)	0.0439 (13)	
H40A	0.9956	1.2235	0.5491	0.053*	
H40B	0.8671	1.2615	0.5391	0.053*	
H40C	0.9738	1.2251	0.4405	0.053*	

### Atomic displacement parameters (Å^2^)

**Table:**

	*U*^11^	*U*^22^	*U*^33^	*U*^12^	*U*^13^	*U*^23^
Rh1	0.0236 (2)	0.0238 (2)	0.0255 (2)	0.00195 (15)	−0.01058 (16)	−0.01111 (15)
Rh2	0.0222 (2)	0.0280 (2)	0.0274 (2)	0.00375 (15)	−0.01155 (16)	−0.01380 (16)
O1	0.0239 (16)	0.0353 (19)	0.0290 (18)	0.0008 (14)	−0.0084 (14)	−0.0137 (15)
O2	0.0254 (17)	0.0290 (17)	0.0373 (18)	0.0026 (13)	−0.0143 (15)	−0.0186 (15)
O3	0.0332 (18)	0.0317 (18)	0.0323 (18)	0.0002 (14)	−0.0155 (15)	−0.0150 (15)
O4	0.0285 (17)	0.0350 (18)	0.0252 (17)	0.0027 (14)	−0.0078 (14)	−0.0125 (14)
N1	0.029 (2)	0.0221 (19)	0.028 (2)	0.0032 (16)	−0.0151 (17)	−0.0080 (16)
N2	0.030 (2)	0.024 (2)	0.032 (2)	0.0030 (16)	−0.0142 (18)	−0.0073 (17)
N3	0.032 (2)	0.023 (2)	0.030 (2)	0.0038 (16)	−0.0122 (18)	−0.0157 (17)
N4	0.0225 (19)	0.030 (2)	0.030 (2)	0.0028 (16)	−0.0112 (17)	−0.0135 (17)
N5	0.024 (2)	0.032 (2)	0.029 (2)	0.0041 (16)	−0.0128 (17)	−0.0147 (17)
C1	0.032 (3)	0.023 (2)	0.038 (3)	0.003 (2)	−0.017 (2)	−0.010 (2)
C2	0.060 (4)	0.034 (3)	0.044 (3)	0.000 (3)	−0.025 (3)	−0.012 (2)
C3	0.022 (2)	0.032 (3)	0.032 (3)	0.003 (2)	−0.009 (2)	−0.011 (2)
C4	0.042 (3)	0.036 (3)	0.028 (3)	−0.004 (2)	−0.009 (2)	−0.007 (2)
C5	0.018 (2)	0.031 (3)	0.035 (3)	0.0058 (19)	−0.010 (2)	−0.012 (2)
C6	0.027 (3)	0.041 (3)	0.040 (3)	0.008 (2)	−0.011 (2)	−0.018 (2)
C7	0.032 (3)	0.027 (2)	0.029 (3)	0.001 (2)	−0.016 (2)	−0.009 (2)
C8	0.028 (3)	0.041 (3)	0.043 (3)	0.006 (2)	−0.015 (2)	−0.021 (2)
C9	0.038 (3)	0.018 (2)	0.028 (2)	0.000 (2)	−0.016 (2)	−0.0025 (19)
C10	0.053 (3)	0.041 (3)	0.045 (3)	0.001 (3)	−0.022 (3)	−0.017 (3)
C11	0.075 (4)	0.044 (3)	0.036 (3)	−0.007 (3)	−0.010 (3)	−0.017 (3)
C12	0.113 (6)	0.043 (3)	0.036 (3)	−0.030 (4)	−0.041 (4)	0.005 (3)
C13	0.083 (5)	0.044 (3)	0.062 (4)	−0.017 (3)	−0.053 (4)	0.006 (3)
C14	0.045 (3)	0.038 (3)	0.046 (3)	−0.003 (2)	−0.026 (3)	−0.006 (2)
C15	0.038 (3)	0.027 (2)	0.030 (3)	0.004 (2)	−0.018 (2)	−0.008 (2)
C16	0.032 (3)	0.035 (3)	0.051 (3)	0.006 (2)	−0.013 (3)	−0.018 (3)
C17	0.047 (3)	0.040 (3)	0.067 (4)	0.014 (3)	−0.020 (3)	−0.027 (3)
C18	0.067 (4)	0.038 (3)	0.057 (4)	0.007 (3)	−0.030 (3)	−0.023 (3)
C19	0.065 (4)	0.032 (3)	0.058 (4)	−0.010 (3)	−0.029 (3)	−0.011 (3)
C20	0.042 (3)	0.035 (3)	0.039 (3)	−0.009 (2)	−0.010 (2)	−0.010 (2)
C21	0.025 (2)	0.033 (3)	0.036 (3)	0.004 (2)	−0.005 (2)	−0.015 (2)
C22	0.027 (2)	0.039 (3)	0.036 (3)	0.004 (2)	−0.014 (2)	−0.022 (2)
C23	0.031 (3)	0.041 (3)	0.030 (3)	0.011 (2)	−0.014 (2)	−0.015 (2)
C24	0.024 (2)	0.034 (3)	0.039 (3)	0.006 (2)	−0.011 (2)	−0.019 (2)
C25	0.042 (3)	0.044 (3)	0.038 (3)	0.004 (2)	−0.017 (2)	−0.020 (2)
C26	0.058 (4)	0.043 (3)	0.035 (3)	−0.005 (3)	−0.022 (3)	−0.012 (2)
C27	0.053 (3)	0.032 (3)	0.043 (3)	−0.005 (2)	−0.018 (3)	−0.015 (2)
C28	0.018 (2)	0.036 (3)	0.032 (3)	0.0073 (19)	−0.009 (2)	−0.015 (2)
C29	0.039 (3)	0.046 (3)	0.051 (3)	−0.005 (2)	−0.026 (3)	−0.010 (3)
C30	0.051 (3)	0.053 (4)	0.039 (3)	0.002 (3)	−0.026 (3)	−0.005 (3)
C31	0.038 (3)	0.077 (4)	0.042 (3)	0.013 (3)	−0.022 (3)	−0.028 (3)
C32	0.035 (3)	0.056 (4)	0.052 (3)	−0.003 (3)	−0.022 (3)	−0.031 (3)
C33	0.031 (3)	0.035 (3)	0.047 (3)	0.001 (2)	−0.018 (2)	−0.014 (2)
C34	0.026 (2)	0.033 (3)	0.025 (2)	0.007 (2)	−0.017 (2)	−0.013 (2)
C35	0.025 (2)	0.042 (3)	0.041 (3)	0.003 (2)	−0.015 (2)	−0.017 (2)
C36	0.040 (3)	0.044 (3)	0.052 (3)	0.010 (2)	−0.024 (3)	−0.028 (3)
C37	0.027 (3)	0.058 (4)	0.051 (3)	0.016 (2)	−0.020 (3)	−0.037 (3)
C38	0.034 (3)	0.068 (4)	0.042 (3)	0.001 (3)	−0.014 (3)	−0.029 (3)
C39	0.031 (3)	0.044 (3)	0.032 (3)	−0.001 (2)	−0.010 (2)	−0.013 (2)
C40	0.039 (3)	0.043 (3)	0.056 (4)	−0.003 (2)	−0.017 (3)	−0.021 (3)

### Geometric parameters (Å, º)

**Table:**

Rh1—O1	2.030 (3)	C11—C12	1.374 (9)
Rh1—O2	2.037 (3)	C12—C13	1.371 (9)
Rh1—N1	2.049 (3)	C13—C14	1.383 (7)
Rh1—N2	2.064 (4)	C15—C20	1.386 (6)
Rh1—N3	2.161 (4)	C15—C16	1.395 (7)
Rh1—Rh2	2.4039 (5)	C16—C17	1.386 (7)
Rh2—O3	2.037 (3)	C17—C18	1.382 (8)
Rh2—O4	2.041 (3)	C18—C19	1.353 (8)
Rh2—N4	2.064 (4)	C19—C20	1.393 (7)
Rh2—N5	2.072 (3)	C21—C22	1.447 (6)
O1—C5	1.285 (5)	C22—C23	1.382 (7)
O2—C7	1.285 (5)	C22—C27	1.388 (7)
O3—C1	1.288 (5)	C23—C24	1.391 (6)
O4—C3	1.294 (5)	C24—C25	1.386 (7)
N1—C1	1.307 (5)	C24—C40	1.505 (7)
N1—C9	1.433 (5)	C25—C26	1.384 (7)
N2—C3	1.317 (6)	C26—C27	1.384 (7)
N2—C15	1.426 (6)	C28—C33	1.379 (6)
N3—C21	1.138 (6)	C28—C29	1.379 (7)
N4—C5	1.318 (5)	C29—C30	1.376 (7)
N4—C28	1.435 (5)	C30—C31	1.382 (8)
N5—C7	1.303 (5)	C31—C32	1.372 (8)
N5—C34	1.441 (5)	C32—C33	1.387 (7)
C1—C2	1.508 (6)	C34—C35	1.387 (6)
C3—C4	1.508 (6)	C34—C39	1.387 (6)
C5—C6	1.503 (6)	C35—C36	1.398 (7)
C7—C8	1.511 (6)	C36—C37	1.363 (7)
C9—C10	1.374 (7)	C37—C38	1.381 (8)
C9—C14	1.376 (7)	C38—C39	1.396 (7)
C10—C11	1.394 (7)		
			
O1—Rh1—O2	178.13 (12)	O1—C5—C6	115.0 (4)
O1—Rh1—N1	88.19 (13)	N4—C5—C6	121.7 (4)
O2—Rh1—N1	91.47 (13)	O2—C7—N5	122.9 (4)
O1—Rh1—N2	89.89 (13)	O2—C7—C8	112.8 (4)
O2—Rh1—N2	90.19 (14)	N5—C7—C8	124.2 (4)
N1—Rh1—N2	171.66 (14)	C10—C9—C14	119.4 (5)
O1—Rh1—N3	94.35 (13)	C10—C9—N1	120.9 (4)
O2—Rh1—N3	87.51 (13)	C14—C9—N1	119.4 (4)
N1—Rh1—N3	94.62 (14)	C9—C10—C11	120.5 (5)
N2—Rh1—N3	93.62 (14)	C12—C11—C10	119.9 (6)
O1—Rh1—Rh2	89.68 (8)	C13—C12—C11	119.3 (5)
O2—Rh1—Rh2	88.46 (8)	C12—C13—C14	121.1 (6)
N1—Rh1—Rh2	85.18 (10)	C9—C14—C13	119.8 (5)
N2—Rh1—Rh2	86.69 (10)	C20—C15—C16	119.0 (4)
N3—Rh1—Rh2	175.96 (10)	C20—C15—N2	122.3 (4)
O3—Rh2—O4	177.98 (12)	C16—C15—N2	118.6 (4)
O3—Rh2—N4	89.74 (14)	C17—C16—C15	120.2 (5)
O4—Rh2—N4	88.47 (13)	C18—C17—C16	120.4 (5)
O3—Rh2—N5	90.82 (14)	C19—C18—C17	119.3 (5)
O4—Rh2—N5	90.82 (14)	C18—C19—C20	121.8 (5)
N4—Rh2—N5	172.16 (14)	C15—C20—C19	119.3 (5)
O3—Rh2—Rh1	90.08 (8)	N3—C21—C22	175.1 (5)
O4—Rh2—Rh1	88.86 (8)	C23—C22—C27	120.9 (4)
N4—Rh2—Rh1	85.65 (10)	C23—C22—C21	117.8 (4)
N5—Rh2—Rh1	86.53 (10)	C27—C22—C21	121.1 (4)
C5—O1—Rh1	117.4 (3)	C22—C23—C24	120.5 (4)
C7—O2—Rh1	119.2 (3)	C25—C24—C23	118.6 (5)
C1—O3—Rh2	117.6 (3)	C25—C24—C40	120.9 (4)
C3—O4—Rh2	118.7 (3)	C23—C24—C40	120.5 (4)
C1—N1—C9	119.9 (4)	C26—C25—C24	120.5 (5)
C1—N1—Rh1	121.5 (3)	C25—C26—C27	121.0 (5)
C9—N1—Rh1	118.1 (3)	C26—C27—C22	118.3 (5)
C3—N2—C15	121.4 (4)	C33—C28—C29	119.4 (5)
C3—N2—Rh1	120.0 (3)	C33—C28—N4	120.4 (4)
C15—N2—Rh1	118.6 (3)	C29—C28—N4	120.2 (4)
C21—N3—Rh1	166.4 (4)	C30—C29—C28	119.9 (5)
C5—N4—C28	119.3 (4)	C29—C30—C31	121.2 (5)
C5—N4—Rh2	119.9 (3)	C32—C31—C30	118.6 (5)
C28—N4—Rh2	120.7 (3)	C31—C32—C33	120.8 (5)
C7—N5—C34	119.9 (4)	C28—C33—C32	120.0 (5)
C7—N5—Rh2	119.4 (3)	C35—C34—C39	119.3 (4)
C34—N5—Rh2	120.6 (3)	C35—C34—N5	119.5 (4)
O3—C1—N1	122.7 (4)	C39—C34—N5	121.2 (4)
O3—C1—C2	114.6 (4)	C34—C35—C36	119.5 (5)
N1—C1—C2	122.6 (4)	C37—C36—C35	121.4 (5)
O4—C3—N2	122.5 (4)	C36—C37—C38	119.1 (5)
O4—C3—C4	115.0 (4)	C37—C38—C39	120.7 (5)
N2—C3—C4	122.5 (4)	C34—C39—C38	119.9 (5)
O1—C5—N4	123.4 (4)		
			
O1—Rh1—Rh2—O3	−75.69 (8)	N1—Rh1—Rh2—O3	12.55 (11)
O1—Rh1—Rh2—O4	102.59 (8)	N1—Rh1—Rh2—O4	−169.17 (11)
O1—Rh1—Rh2—N4	14.04 (8)	N1—Rh1—Rh2—N4	102.28 (11)
O1—Rh1—Rh2—N5	−166.52 (8)	N1—Rh1—Rh2—N5	−78.28 (11)
O2—Rh1—Rh2—O3	104.11 (8)	N2—Rh1—Rh2—O3	−165.59 (11)
O2—Rh1—Rh2—O4	−77.62 (8)	N2—Rh1—Rh2—O4	12.69 (11)
O2—Rh1—Rh2—N4	−166.17 (8)	N2—Rh1—Rh2—N4	−75.86 (11)
O2—Rh1—Rh2—N5	13.28 (8)	N2—Rh1—Rh2—N5	103.59 (11)
